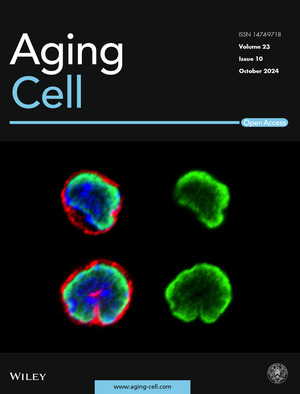# Additional Cover

**DOI:** 10.1111/acel.14380

**Published:** 2024-10-09

**Authors:** Sachin Kumar, Jeffrey D. Vassallo, Kalpana J. Nattamai, Aishlin Hassan, Angelika Vollmer, Rebekah Karns, Mehmet Sacma, Travis Nemkov, Angelo D'Alessandro, Hartmut Geiger

## Abstract

Cover legend: The cover image is based on the Article *Rejuvenation of the reconstitution potential and reversal of myeloid bias of aged HSCs upon pH treatment* by Sachin Kumar et al., https://doi.org/10.1111/acel.14324